# Regulated Entry of Hepatitis C Virus into Hepatocytes

**DOI:** 10.3390/v9050100

**Published:** 2017-05-09

**Authors:** Zhijiang Miao, Zhenrong Xie, Jing Miao, Jieyu Ran, Yue Feng, Xueshan Xia

**Affiliations:** 1 Faculty of Life Science and Technology, Kunming University of Science and Technology, Kunming 650500, China; miaozhijiang@yeah.net (Z.M.); sanyue2016@yahoo.com (J.M.); R6535930@126.com (J.R.); 2 Yunnan Institute of Digestive Disease, the First Affiliated Hospital of Kunming Medical University, Kunming 650032, China; xiezhenrong2006kycg@126.com

**Keywords:** hepatitis C virus, cell entry, regulation, signaling, receptor

## Abstract

Hepatitis C virus (HCV) is a model for the study of virus–host interaction and host cell responses to infection. Virus entry into hepatocytes is the first step in the HCV life cycle, and this process requires multiple receptors working together. The scavenger receptor class B type I (SR-BI) and claudin-1 (CLDN1), together with human cluster of differentiation (CD) 81 and occludin (OCLN), constitute the minimal set of HCV entry receptors. Nevertheless, HCV entry is a complex process involving multiple host signaling pathways that form a systematic regulatory network; this network is centrally controlled by upstream regulators epidermal growth factor receptor (EGFR) and transforming growth factor β receptor (TGFβ-R). Further feedback regulation and cell-to-cell spread of the virus contribute to the chronic maintenance of HCV infection. A comprehensive and accurate disclosure of this critical process should provide insights into the viral entry mechanism, and offer new strategies for treatment regimens and targets for HCV therapeutics.

## 1. Introduction

Hepatitis C virus (HCV) is a hepatotropic member of the *Flaviridae* family. HCV causes chronic hepatitis, which may result in tissue damage, fibrosis, cirrhosis, and the eventual development of hepatocellular carcinoma (HCC) [1]. HCV particles are enveloped by covalently linked envelope glycoproteins 1 and 2 (E1 and E2) [2]. Viral particles produced by hepatocytes are typically found in association with various lipids and apolipoproteins (Apo), such as ApoA1, ApoB, ApoC1/2/3, ApoE, and cholesterol, as lipid-rich particles known as lipoviroparticles (LVPs) [3,4,5,6]. ApoE appears to play a particularly important role for the formation and function of these LVPs [7,8,9,10,11,12]. Because of this association, LVPs display a broad buoyant density profile, and the majority of infectious viral RNA in the plasma of infected patients coelute with very low-density lipoproteins (VLDLs) [3,13,14,15]. 

Since the discovery of HCV in 1989 [16], several experimental systems have been developed for the study of HCV, and numerous functional genomic and proteomic studies have been performed [17,18,19,20,21]. These studies showed that HCV entry into hepatocytes involves a complex multi-step process that engages various cellular proteins. Previous studies suggested that heparan sulfate proteoglycans (HSPGs) [22], low-density lipoprotein receptor (LDLR) [23], SR-BI [24], CD81 [25], EGFR [26], and two tight junction proteins—namely claudin-1 (CLDN1) [27] and occludin (OCLN) [28]—as well as several other host factors, are sufficient to support HCV entry [28]. However, new putative entry-related factors are continuously being identified and characterized, such as dendritic cell-specific intercellular adhesion molecule 3-grabbing nonintegrin (DC-SIGN), liver/lymph node-specific intercellular adhesion molecule-3-grabbing integrin (L-SIGN) [29,30,31], Niemann-Pick C1-Like 1 (NPC1L1) [32], transferrin receptor 1 (TfR1) [33], and mothers against decapentaplegic homolog 6 and 7 (SMAD6/7) [20,34]. Additionally, the interaction between these factors, their functions in related regulatory pathways, and how they function in a coordinated manner, remain to be further elucidated; these are reviewed in this article. Because virus entry into host cells is the first step in the HCV life cycle, a comprehensive and accurate evaluation of the virus entry process should provide further insights into the mechanism, and offer paths for new treatment regimens and targets for HCV therapeutics.

## 2. Cell-Free Entry

### 2.1. Before Binding

#### 2.1.1. Virus Landing

**Figure 1:**
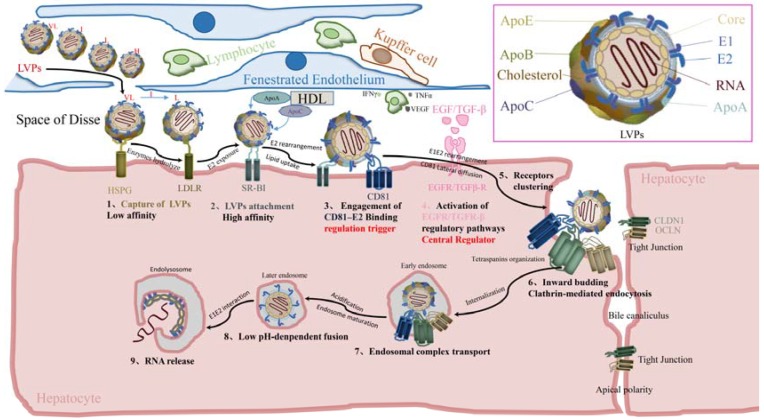
Proposed model of hepatitis C virus (HCV) entry into hepatocytes. The microstructure of the space of Disse of the liver is shown in the top-left corner. Foreign HCV particles are tightly associated with lipoproteins to form a complex termed lipoviroparticles (LVPs). Incorporated lipoproteins of varying densities from very low (VL), intermediate (I), and low (L) to high (H) are shown in the top right corner. LVPs are typically transported by sinusoidal blood flow into the space of Disse through fenestrated endothelium. The different HCV receptors, co-factors, and host cell components involved in HCV entry are shown. Major HCV entry steps are presented in bold text (from 1 to 9). Between the steps, relevant changes or interactions are indicated with arrows either above or below, indicating the direction of pathways involved in LVP entry. Upon their arrival in the space of Disse, infectious LVPs, which typically contain VLDL, are initially captured by HSPG and LDLR. This step involves sequential enzymatic hydrolyses by LPL and hepatic lipase to convert VLDL to LDL, via its intermediate form. Captured LVPs subsequently interact with SR-BI, owing to their high affinity to exposed E2 and CEs. Here, SR-BI mediates the transfer of ApoA and ApoC1 from HDL to the particles and causes E2 conformation change. This SR-BI-mediated change enables the particles to access and bind to CD81, inducing E1E2 conformational change. The engagement of CD81 triggers the sequential regulations that are centrally controlled by EGFR and TGFβ-R; most of these regulatory mechanisms are involved in subsequent processes leading to CD81 migration to tight junctions. Tight junction proteins CLDN1 and OCLN can then interact with the complex, likely via an OCLN–CLDN1 EL1–CD81 LEL molecular interface, leading to the clustering of tetraspanins. Thereafter, entry complexes clustered in tight junctions are internalized through inward budding and clathrin-dependent endocytosis; these processes are also controlled by multiple regulatory pathways. The primary endocytic vesicles are converted from early endosomes to late endosomes, and the maturation process is facilitated by RAB5A-mediated acidification. Following endosome acidification, virus particles are exposed to an acidified environment, which induces additional, irreversible conformational changes. Further, fusion is triggered by the insertion of fusion peptide into the late endosomal membrane. Late endosomes encounter and merge with lysosomes to form endolysosomes, which are then converted to the classical dense lysosomes, resulting in the ultimate release of viral genome into the cell cytosol. Abbreviations: VLDL, low-density lipoproteins; HSPG, heparan sulfate proteoglycan; LDLR, low-density lipoprotein receptor; LDL, low-density lipoprotein; CEs, cholesteryl esters; ApoA, apolipoprotein A; ApoC1, apolipoprotein C1; HDL, high-density lipoprotein; CD81, cluster of differentiation 81; EGFR, epidermal growth factor receptor; TGFβ-R, transforming growth factor β receptor; CLDN1, claudin-1; OCLN, occluding; LEL, large extracellular loop; RAB5A, Ras-related protein Rab-5A.

HCV is a blood-borne infectious agent that only infects human and chimpanzees naturally. Within the host, though HCV can replicate to low levels in non-hepatic cells, such as cells derived from brain tissue [35], polarized hepatic cells are the primary platform for HCV landing and, unsurprisingly, express the full complement of entry receptors. The liver comprises mainly of hepatocytes, and also contains non-parenchymal cells such as endothelial, Kupffer, stellate cells, and lymphocytes [36]. Hepatocytes are structurally and functionally polarized with three distinct membrane domains: the sinusoidal (basal), lateral, and canalicular (apical) surfaces [37,38]. The sinusoidal endothelium is highly fenestrated and closely associated with hepatocytes and stellate cells in the space of Disse. Further DC-SIGN is expressed on some dendritic cells (DCs), while L-SIGN is abundantly expressed on sinusoidal endothelial cells. Many previous studies have showed that DCs may bind multiple pathogens via DC-SIGN at a site of mucosal exposure and carry the virus to target cells within the draining lymph node, thereby facilitating establishment of an infection [39]. Other studies showed that L-SIGN and DC-SIGN specifically bind HCV envelope glycoprotein E2 [29,31], and this binding of HCV E2 to immature DCs was dependent on DC-SIGN interactions [30]. This suggests that L-SIGN and DC-SIGN initially interact with HCV and deliver the virus to the liver target cells, which may explain HCV tissue tropism and contribute to the establishment or persistence of infection. Thus, within the liver, LVPs are transported by sinusoidal blood flow, through molecular sieve fenestration formed by the endothelium, and into the space of Disse, where the virus can be in contact with receptors on the basal membrane of hepatocytes (Figure 1).

#### 2.1.2. Capture of Viral Particles

Once HCV LVPs are present in the space of Disse, where the basolateral membrane of hepatocytes are exposed, LVPs are captured by HSPG and LDLR on the surface of hepatocytes in a spatiotemporally regulated manner. HSPG, namely heparin/heparan (HSGAG), is a particular glycosaminoglycan (GAG) that plays an important role during HCV entry. HSPG is abundant in the space of Disse and can mediate the metabolism of remnant lipoproteins [40]. The intestinally derived remnant lipoproteins are initially sequestrated within the space of Disse, where ApoE secreted by hepatocytes enhances remnants binding to HSPG and uptake. Subsequently, the remnants undergo further processing by hepatic and lipoprotein lipases (LPL). In parallel, nascent VLDL particles released into the plasma, which are not ligands for LDLR, must be first processed by LPL. LPL hydrolyzes triglycerides and removes 70% of the resulting intermediate-density lipoproteins (IDLs). The remaining IDLs are converted to LDLs, which are rich in cholesteryl esters (CEs) but have only ApoB as the sole apolipoprotein. Thereafter, hepatic lipases catalyze reactions to further reduce the triglyceride amount [41]. VLDL particles, which contain ApoC1 and ApoC2, are incorporated into LVPs. ApoC1 can interact with HSPG, but it inhibits the binding of lipoprotein to LDLR or VLDL receptor (VLDLR), and blocks CE transfer [2]. ApoC2 is the physiological activator of lipases and promotes the clearance of triglyceride-rich lipoproteins (TRLs) [42]. These facts suggest that LVPs containing ApoC1 are not initially able to interact with LDLR; the interaction between LDLR and ApoB is possible only after enzymatic hydrolysis. 

Taken together, LVPs near to the surface of hepatocytes are initially captured by HSPG via the interaction of ApoE and ApoC1 with HSPG. Here, ApoE mainly interacts with HSPG proteins syndecan (SDC) 1 and 4, although the interaction with SDC4 has a more prominent effect [43,44]. LVPs are sequentially hydrolyzed by LPL and hepatic lipase, resulting in ApoB exposure, which enables its interaction with LDLR. 

#### 2.1.3. Viral Particles Attachment

SR-BI is a cell surface receptor for high-density lipoprotein (HDL) found in various cell types [45,46]. It is expressed in high levels in the liver and steroidogenic tissues [47]. SR-BI mediates lipid metabolism, particularly the uptake and recycling of HDL particles, and binds to different classes of lipoproteins, such as VLDL, LDL, and HDL [48]. Additionally, SR-BI can mediate the transport and endocytosis of CEs (which is esterified from plasma HDL-cholesterol by LPL) into the liver [49]. Considering that after LVP interaction with LDLR, CEs are enriched in LDL as the main lipoprotein component, SR-BI may act as the receptor subsequent to LDLR; here, CEs are likely to interact with SR-BI. Several studies have demonstrated that the lipid transfer activity of SR-BI (SR-BI mediates the transfer of ApoA and ApoC1 from HDL to LVPs) [50,51,52] mediates the HCV entry, but not the E2–SR-BI interaction (possibly through interaction with E2 hypervariable region 1 [53]. However, the hypervariable region 1 (HVR1), a 27 amino acid segment located at the N-terminus of the E2 glycoprotein, obstructs the viral CD81 binding site and conserved neutralizing epitopes, and deletion of HVR1 impairs the binding of soluble E2 to human hepatoma cells [24,25,54,55]. The lipid transfer activity of SR-BI has been identified as an “access” function; blocking such “access” function also inhibits the entry of HCV cell culture (HCVcc) particles of all densities [54]. Furthermore, the “access” function of SR-BI can modify the lipoprotein profile of virus particles and simultaneously enrich the cellular membrane for cholesterol. Concurrent with lipoprotein rearrangements on HCV particles, hidden E1E2 epitopes may be exposed, which enable E1E2 binding to other receptors [54], as demonstrated by the binding of soluble E2 proteins, but not HCV particles, to CD81 [56,57]. This suggests that HCV–SR-BI interaction alters the conformation of HCV virions [58,59]. Taken together, the attachment of HCV virus particles is mediated by E2 and requires the lipid transfer activity of SR-BI, leading to E2 conformational change. 

### 2.2. E2–CD81 Binding and Activation of Regulatory Pathways

#### 2.2.1. Engagement of E2–CD81 Binding

Following their attachment to SR-BI, early lipoprotein rearrangement of the particles exposes receptor binding sites in E2 glycoprotein, which allows for the subsequent binding of virus particles to CD81 tetraspanin. Human CD81 is broadly expressed and involved in various cellular functions such as adhesion, morphology, proliferation, and differentiation. CD81 has two extracellular domains, the small extracellular loop (SEL) and large extracellular loop (LEL), and is anchored to the cell membrane through its four transmembrane domains [60]. The HCV-CD81 binding involves an interaction between the CD81 LEL (E2-binding site on CD81 LEL, including amino acids 113 to 201, comprises residues Leu^162^, Ile^182^, Asn^184^ and Phe^186^) [61], and conformational region of E2 (including the downstream of the second hypervariable region 480–493, as well as amino acids 544–551 and 612–619, along with several individual residues Trp^420^, Tyr^527^, Trp^529^, Gly^530^ and Asp^535^) [62,63,64]. Further, a mutagenesis analysis identified a particular crosstalk between three amino acids of E1 and the domain III of E2. Such crosstalk leads to either a full restoration of the functionality of the suboptimal heterodimer, or a destabilization of the functional heterodimer, which thus modulates E1E2 binding to SR-BI and CD81 [65]. The above suggests that CD81 can interact with E2 during CD81 binding, and the engagement of CD81 can induce further E1E2 conformational rearrangement, which primes HCV for internalization and low pH-dependent fusion.

#### 2.2.2. Activation of Regulatory Pathways

Non-parenchymal cells in the liver can regulate the distribution of virus receptors, thereby influencing HCV dissemination in the liver through the expression of cytokines and growth factors activated by inflammation [36,66]. The glycosaminoglycan heparan sulfate (HS) can act as a co-receptor for various polypeptide growth factors, especially members of the epidermal growth factor (EGF) family and transforming growth factor (TGF) α and β [66]. Members of the TGFβ superfamily play a crucial role in regulating cellular growth and differentiation in a wide range of biological systems [67]. Additionally, tetraspanins can serve as scaffolds in various signaling processes to organize membrane-related proteins and other tetraspanin family members into a specific, highly organized network [38]. HCV E2–CD81 binding triggers the phosphorylation of EGFR and TGFβ-R, leading to the activation of several regulatory pathways and signaling cascades—these regulatory pathways are involved in subsequent HCV post-binding entry processes (see the “Regulatory Mechanisms for HCV Entry” section below).

### 2.3. Postbinding

#### Lateral Diffusion of CD81 and Receptor Clustering

The two tight junction proteins, CLDN1 and OCLN, likely act following the binding of CD81 to SR-BI [27,28,68,69]. Like CD81, both CLDN1 and OCLN are tetraspanins with four transmembrane domains, in addition to an LEL and SEL (termed EL1 and EL2, respectively). Interestingly, CLDN1 is incorporated as a component of the HCV receptor complex for its interaction with CD81; however, it does not directly interact with HCV particles [70,71]. Furthermore, CD81–CLDN1 complexes are found near intercellular junctions during HCV infection of Huh7.5 cells, which is likely mediated through a CLDN1 EL1–CD81 LEL molecular interface [70,72,73]. Following CD81 binding to CLDN1, the HCV receptor complex is thought to interact with OCLN and internalize at cellular tight junctions [74]. Synchronized infection assays showed that OCLN downregulation can decrease both HCV entry and glycoprotein-mediated cell fusion. It has been suggested that OCLN EL2 is involved in HCV pseudoparticles (HCVpp) entry, as well as in E2 binding [47]. Indeed, the interaction between E2 and OCLN has been demonstrated using confocal microscopy and coimmunoprecipitation assays [69,75,76]. Additionally, like CD81 LEL, six residues located within OCLN EL2 have been shown to mediate HCV host tropism [28,77]. However, OCLN does not appear to interact directly with HCV particles, akin to its role in mediating the entry of coxsackie virus B (CVB) into target cells without directly interacting with the virus [78]. Instead, OCLN likely functions with CLDN1 to enable HCV entry; importantly, like CD81, OCLN must be of a human origin to overcome the host species barrier [28].

Nevertheless, hepatocytes are highly polarized and display a unique distribution of tight junction proteins on the plasma membrane. CLDN1 localizes at basolateral and apical hepatocyte membranes in nonpolarized liver tissues, but is predominantly found at the apical bile canalicular membrane surface in polarized hepatocytes [36,37,79,80]. And OCLN is predominantly expressed at the apical membrane of hepatocytes in the liver [81]. Therefore, both CLDN1 and OCLN are predominantly expressed at the apical surface, but are insufficient at the basolateral surface in polarized hepatocytes. Thus, they are spatially inaccessible to HCV particles. This suggests that virus–CD81 complexes will need to be delivered to the areas of cell-to-cell contact, where they can come into contact with tight junction proteins [38]. Such receptor clustering has been shown to be critical for the subsequent virus particle internalization [47]. Additionally, tetraspanins often move laterally along the plasma membrane [38]. The translocation of engaged CD81 molecules requires an intact actin network [38] and appears to occur only when sufficient number of receptors are engaged to initiate the signaling events required for internalization [82]. The lateral diffusion of CD81 likely enhances HCV association with cell receptor complexes, thereby promoting virus migration toward tight junctions [47]. This lateral migration of bound virus mediated by CD81 leads to the relocalization of HCV particles from the basolateral surface, to tight junctions of polarized hepatocytes [38]. 

In summary, CD81 binding may activate the receptor tyrosine kinase (RTK) pathway, leading to actin rearrangements that allow CD81 lateral diffusion within the plasma membrane. This event promotes the stable clustering of receptor proteins at the tight junction region of cells facing the bile canaliculi.

### 2.4. Internalization

#### 2.4.1. Formation of Primary Endocytic Vesicles

The clustering of tetraspanins at tight junctions induces clathrin- and actin-mediated endocytosis [72]. The endocytosis pathway involves various factors associated with clathrin coat components (CLTB, clathrin, light chain B; CLTCL1, clathrin heavy polypeptide-like 1; HIP1, huntingtin-interacting protein 1; HIP1R, huntingtin-interacting protein 1-related protein), activating protein 2 (AP-2)-clathrin recruitment (SYT1, synaptotagmin-1), clathrin and actin cytoskeleton linking (EPN1, epsin-1; EPN3, epsin-3), actin polymerization (CFL1, cofilin 1; Cdc42, cell division control protein 42 homolog; ROCK2, Rho associated coiled-coil containing protein kinase 2), and E3 ubiquitin ligase (c-Cbl, casitas B-lineage lymphoma, which is involved in receptor ubiquitination and RTK signaling) [83]. Previous studies have demonstrated that several viruses can undergo retrograde transport from either actin-rich filopodia or retraction fibers to the cell surface, where entry receptors are clustered, before being internalized [84,85,86,87,88]. The cortical actin cytoskeleton is plastic for the cell, but may act as a barrier for viruses. To overcome this barrier, many viruses trigger the breakdown of actin stress fibers [89,90]. Studies have found that HCV is associated with or transported along intact stress fibers—additionally, an increase in stress fibers has been observed during HCV infection [38]. In a previous study, single-particle tracking was used to investigate the internalization process of virus particles. The results showed that virus internalization can be divided into the following steps: (1) the inward budding of the plasma membrane and the formation of a clathrin pit induced by particle attachment [47]; (2) the ubiquitination of a co-receptor molecule by c-Cbl upon the association of virus-receptor complexes with clathrin-coated pits, which is essential for the subsequent internalization and sorting into endosomes; (3) actin nucleation; and (4) the induction of clathrin-coated pit curvature and deformation by specialized adapter molecules epsins 1 and 3, and the linkage of clathrin coat to actin cytoskeleton, leading to membrane fission and endocytosis [84].

Taken together, when virions retract along filopodia to reach the cell body, HCV-receptors complex forms primary endocytic vesicles via multiple, sequential interactions of HCV virions with both clathrin and actin cytoskeleton. These include the initial formation of a clathrin pit, the subsequent receptor ubiquitination and actin nucleation, and lastly, the induction of clathrin-coated pit curvature and deformation, leading to membrane fission and endocytosis [84].

#### 2.4.2. Signal Degradation and Endosome Maturation

Primary endocytic vesicles undergo the endosomal maturation process, resulting in the delivery of vehicle contents and membranes to the peripheral early endosome (EE), which is ultimately converted to late endosome (LE) [91]—the hepatocyte growth factor-regulated tyrosine kinase substrate (Hrs; encoded by the HGS gene) plays an important role in this process. Hrs on early endosome membrane is phosphorylated by activated tyrosine kinase receptors (TKRs), which are enriched in primary endocytic vesicles [92]. Phosphorylated Hrs can then induce localized invagination of endosomal membrane. Ubiquitinated receptors are sorted into the invagination by either a direct interaction with the ubiquitin interacting motif (UIM) of Hrs, or an indirect interaction with Hrs-binding proteins, such as sorting nexin-1 (SNX1), clathrin, or epidermal growth factor receptor substrate 15 (Eps15) [93,94,95,96]. Then, the membrane ispinched-off to form multivesicular bodies (MVBs). The formation of MVBs leads to the reversal of membrane topology, and consequently, the cytoplasmic portion of TKRs is sorted into internal vesicles of the MVBs, causing TKR inability to signal to downstream components [96]. This event leads to the degradation of active TKRs and downregulation of TKR signaling.

Prior to its conversion to LE, EE needs to accumulate cargo and support membrane recycling or degradation. This process takes approximately 8 to 15 min to complete, which is consistent with a previous study that showed a delay of 30 min for HCV entry into EE [97]. Subsequently, nascent LE traffics toward the microtubule organizing center (MTOC), assisted by kinesin and dynein motor proteins. During this process, LE grows in size, acquiring additional intralumenal vesicles (ILVs), and prepares for the fusion with lysosomes. Ultimately, LE arrives in the perinuclear region of the cell, where it encounters lysosomes and undergoes further maturation steps to deliver the mixture of endocytic and secretory components to lysosomes. The fusion process leads to the generation of endolysosomes, a transient hybrid organelle, in which active degradation can take place. The endolysosomes are then converted to the classical dense lysosomes [91,96].

### 2.5. Fusion

#### 2.5.1. Endosome Acidification

An acidification step is required for certain viruses to trigger the conformational change of the fusion protein and merging of membranes [47]. These viruses remain inside the endosome during endosome maturation, until a low internal pH is achieved to trigger the fusion of viral envelope and endosomal membrane. This acidification is facilitated by a vacuolar (H+)-ATPase (V-ATPase), ATP6v0a1 [91]. Ras-related protein Rab-5A (RAB5A), which is involved in the recruitment of RAB7A to late endosomes, is enriched in early endosomes. RAB5A drives the maturation of endosomes by transporting ATP6v0a1 from the *trans*-Golgi network to endocytic vesicles [98]. In previous studies, bafilomycin A1, which affects the endosomal acidic environment by preventing reacidification, was shown to block HCV infection [97,99]. This finding demonstrated that the release of viral RNA through membrane fusion is dependent on endosomal acidification [47]. However, mild acidification is sufficient to trigger fusion—despite the broad pH range for HCVcc fusion, the pH is shifted toward lower values from pH 6.3 to 4.0.

#### 2.5.2. Fusion of Viral Envelope with Endosome Membrane

Enveloped viruses typically release their genetic materials into the cell cytosol by inducing fusion of their envelope with the endosome membrane. HCV utilizes diverse mechanisms to trigger structural and conformational changes of its fusion protein [100]. Following endosome acidification, viral particles are exposed to an acidified environment, which induces additional and irreversible conformational changes to fusion-primed heterodimers. These rearrangements are thought to expose the putative E1 fusion peptide [47]. As observed in alphaviruses, during fusion, low-pH environment causes the disorganization of the distal domain A (DA), a domain that conceals the E1 fusion peptide, thereby unmasking the E1 fusion peptide [47]. Indeed, the HCV envelope glycoproteins E1 and E2 contain potential fusion domains, and HCV E2 has been shown to be required for liposome–HCVcc fusion in vitro [101]. Both HCV and pestiviruses require a post-attachment priming step to ensure the appropriate fusion process [99,102]. Following internalization, a stable fusion-primed heterodimer conformation is achieved through its insertion within the endosomal membrane [99]. 

Taken together, the fusion mechanism can be divided into the following four steps [47]. (1) Fusion proteins undergo conformational changes, which is then followed by endosomal acidification that leads to the unmasking of E1 fusion peptide. (2) Fusion peptide is inserted into the endosomal membrane, which subsequently initiates the fusion process. (3) The external membrane leaflets undergo a lipid mixing during hemifusion. Based on studies of bovine viral diarrhea virus (BVDV-1) entry, the merging of viral envelope and endosomal membrane is thought to be mediated by the refolding of the entire heterodimer complex through an interdependent, drastic structural rearrangements of both E1 and E2 [47]. Interestingly, E1–E2 coevolution networks are critical for E1E2-mediated fusion, and the back-layer domain of E2 has been shown to be a critical effector of global E1E2 refolding during membrane fusion [65]. Following the insertion of the fusion peptide, this domain may be critical for the structural rearrangement of E2 and the subsequent dramatic fold-back of E1. Such fold-back permits membranes to be in close proximity and undergo a subsequent lipid mixing. (4) A complete fusion of the two membranes ultimately unifies the endosome and virus membranes. Following membrane integration, the viral genome is released into the endolysosome and subsequently enters the cytoplasm. Once in the cytosol, the genome is delivered to “membranous web”, where viral translation initiation is thought to take place. Thus, at this point, HCV has successfully entered the cell and switches to the next step of its life cycle.

## 3. Regulatory Mechanisms for HCV Entry

### 3.1. Regulation for CD81 Lateral Diffusion and Receptors Clustering

**Figure 2:**
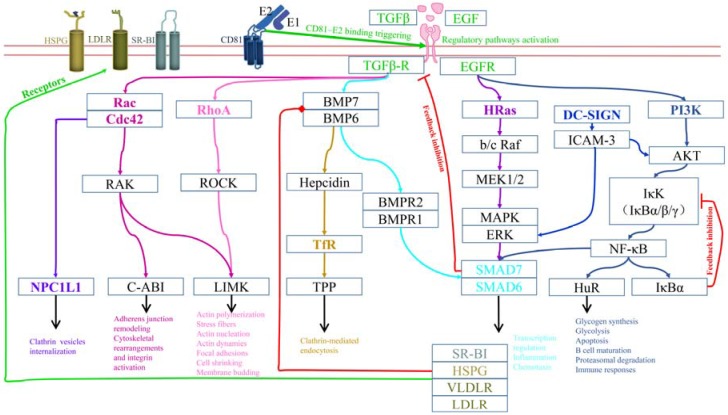
A proposed regulatory signaling network for HCV entry. Signaling activation and entry receptors are indicated in the figure. Only the factors that are related to HCV entry are shown. Relationships between upstream and downstream factors are not limited to regulation; certain downstream factors may serve as a receptor for upstream factors. Specific colors indicate different pathways, while arrows and red lines indicate the main process of a pathway and an inhibitory feedback regulation, respectively. The outcome is shown at the end of each pathway. This network highlights that HCV entry is centrally regulated by EGFR and TGFβ-R. Abbreviations: NPC1L1, Niemann-Pick C1-*l*ike 1; Cdc42, cell division control protein 42 homolog; RhoA, Ras homolog gene family, member A; ROCK, Rho-associated protein kinase; LIMK, LIM domain kinase ; BMP, bone morphogenetic protein; BMPR, bone morphogenetic protein receptor; TfR, transferrin receptor 1; MEK, *m*itogen-activated protein kinase kinase; MAPK, mitogen-activated protein kinase; ERK, extracellular signal-regulated kinase; SMAD6/7, mothers against decapentaplegic homolog 6 and 7; DC-SIGN, dendritic cell-specific intercellular adhesion molecule 3-grabbing nonintegrin; ICAM3, intercellular adhesion molecule 3; PI3K, phosphatidylinositide 3-kinases; AKT, protein kinase B (PKB); IκK, IκB kinase; NF-κB, nuclear factor κ-light-chain-enhancer of activated B cells; HuR, human antigen R; SR-BI, scavenger receptor class B type I.

CD81 binding to HCV E2 induces the activation of multiple EGFR/TGFβ-R regulatory pathways [67,103,104,105] (Figure 2), which manage the CD81 lateral diffusion and receptor clustering processes. CD81 engagement activates various signaling pathways related to GTPases Rac, Rho, and Cdc42, which function together to promote both cytoskeletal rearrangement and actin polymerization [36,38]. Furthermore, the phosphatidylinositide 3-kinases (PI3K)/ protein kinase B (AKT) pathway is also transiently activated [106,107]; because this pathway is involved in glycolysis, it may serve as an energy source for CD81 translocation to tight junctions in an actin-dependent manner. Additionally, the transforming protein p21 (HRas)/mitogen-activated protein kinase (MAPK) pathway is activated in this process [108,109], which induces the clustering of receptor-related tetraspanins and the subsequent compartmentalization of receptors. Thus, cytoskeletal rearrangement and actin polymerization with energy supplementation, combined with the clustering and compartmentalization of tetraspanins, enable CD81 to migrate to tight junctions, and receptors to cluster together.

### 3.2. Regulation of Particle Internalization and Endosome Maturation

In addition to benefiting the CD81 migration, activation of the Rho pathway also promotes particle internalization because it induces actin nucleation and membrane budding. The NPC1L1 pathway is also involved in this process, owing to its role in the internalization of clathrin vesicles and cholesterol uptake [32,110,111,112,113]. Furthermore, HCV infection is associated with iron accumulation in hepatocytes in vivo, and iron uptake is regulated by the TfR pathway, which is mainly involved in clathrin-mediated endocytosis during HCV internalization [33,114]. Most importantly, c-Cbl can selectively ubiquitinate specific receptors [84], and the Hrs pathway can only sort ubiquitinated receptors into the appropriate compartments, a process that is essential for subsequent internalization steps [96]. Additionally, during endosome maturation, a RAB5A-related pathway contributes to the regulation of acidification [91,115,116,117].

### 3.3. Other Regulatory Mechanisms

Previous studies have demonstrated that the nuclear factor κ-light-chain-enhancer of activated B cells (NF-κB) pathway, which regulates gene transcription, cytokine production, and cell survival, is involved during HCV infection. This pathway is also known to be regulated by EGFR. Upon the activation of NF-κB signaling, the NF-κB inhibitor IκBα is phosphorylated by IκB kinase (IKK) via a regulatory domain in IκB. Following its phosphorylation, IκB is also ubiquitinated, leading to its degradation. IκB degradation allows the NF-κB complex to freely enter the nucleus, where it can activate the expression of specific genes, especially those related to inflammation and/or immune responses. However, NF-κB also induces the expression of IκBα, its own repressor, leading to the re-inhibition of NF-κB by de novo synthesized IκBα, forming an auto feedback loop that results in oscillating levels of active NF-κB [118]. NF-κB is known to regulate the inflammatory response. As an example, during human immunodeficiency virus (HIV) infection, NF-κB was reported to regulate the expression of viral genes, thereby influencing its virulence. In this case, NF-κB activation may partly activate the virus from its latent, inactive state [119]. As mentioned above, NF-κB can also regulate the immune response. During a *Yersinia pestis* infection, the bacteria secrete YopP factors to prevent the ubiquitination of IκB, effectively repressing the NF-κB pathway and blocking the immune response to *Y. pestis* [120]. Upon HCV entry, NF-κB also plays an important role. Two recent studies showed that NF-κB could be activated by several tumor necrosis factor (TNF) superfamily members (including TNF-α, TNF-β, TWEAK and LIGHT), which then mediate the activation of myosin light chain kinase (MLCK) [121,122]. The TNF–NF-κB–MLCK pathway can induce the relocalization of OCLN and increase the lateral diffusion speed of CD81 in polarized HepG2 cells. This leads to the disruption of tight junctions and thus increases permissivity to support the entry of HCV. Further, another study showed that activated NF-κB can increase the production of interleukin-1β (IL-1β) by inducing IL-1β messenger RNA (mRNA) expression. The production of IL-1β in turn drives proinflammatory cytokine, chemokine, and immune-regulatory gene expression networks linked with HCV disease severity [123].

A recent study showed that SMAD6 and SMAD7 act as central regulators of HCV entry, a role that is related to the expression of host receptors, such as HSPG, LDLR, VLDLR, and SR-BI. SMAD6 and SMAD7 belong to the inhibitory SMAD (I-SMAD) family, and can negatively regulate the TGFβ signaling pathway through feedback loop mechanisms [124]. A previous study showed that SMAD6/7 overexpression increases the expression of HCV receptor genes; conversely, their knockdown reduces the transcription of these genes [34]. The study also indicated that HCV-infected cells upregulate the expression of SMAD6/7, which requires the activity of NF-κB. Interestingly, SMAD7 is an inhibitor of the TGFβ-R superfamily signaling, and can be induced by bone morphogenetic protein (BMP) through an inhibitory feedback loop [124]. BMP can bind to its co-receptor HSPG, and such binding is required for BMP-7-mediated cellular signaling. However, another study showed that HSPG is only able to mediate BMP signaling when anchored on plasma membrane, and the addition of exogenous heparin prevents BMP-7-mediated SMAD phosphorylation [125]. Another study showed that the specific binding of BMP-6 to heparin induces SMAD1/5/8 phosphorylation, which can be inhibited by heparin [126]. The above indicate that SMAD6/7 pathway is very complex, and involves multiple HCV entry related pathways to regulate receptor genes expression. 

Taken together, both NF-κB and SMAD6/7 pathways regulate the expression of HCV entry-related host receptor genes. In particular, SMAD6/7 molecules are critical regulators of various downstream transcription regulation pathways. The regulation of both NF-κB and SMAD6/7 are associated with physiological responses. Both SMAD6/7 and NF-κB activations can lead to the induction of inflammatory responses; however, NF-κB is also involved in the regulation of immune responses. Both pathways are regulated by inhibitory feedback mechanisms, which may be associated with the effects or consequences of viral infection.

## 4. Cell-to-Cell Spread

### 4.1. Cell-to-Cell Spread Shares the Same Receptors with Cell-Free Entry

HCV can infect naïve hepatocytes through two different means: cell-free entry and cell-to-cell spread. In cell-free entry, extracellular virus particles, which are typically transported through the blood, interact with multiple receptors to enter hepatocytes. Thus, this route of entry typically takes place during the initial infection of the host with HCV. In contrast, cell-to-cell spread or transmission occurs when infected cells transmit viruses to neighboring cells—thus, it mainly takes place in chronically infected hosts [127]. The presence of cell-to-cell spread of HCV is demonstrated through HCV infection of Huh7.5 hepatoma cells, which results in focal areas of infection where transmission is potentiated by cell-cell contact. Additionally, co-culture of HCV-infected hepatoma cells with naïve cells in the presence of monoclonal antibodies or immunoglobulins isolated from infected individuals resulted in the eventual infection of the naïve cells, providing further evidence of HCV cell-to-cell spread [128]. A subsequent study also showed that SR-BI and CLDN1, as well as human CD81 and OCLN, are sufficient for HCV infection of murine cell lines—thus, these molecules constitute the minimal HCV entry receptor set [28]. Importantly, in both cell-free and cell-to-cell spread, HCV utilizes common factors to enter hepatocytes [127]. However, SR-BI has a more prominent role during cell-to-cell transmission, as demonstrated by the preferential blockage of HCV cell-to-cell transmission using SR-BI-specific antibodies, or small-molecule inhibitors [129]. 

### 4.2. Cell-to-Cell Spread Leads to Immune Evasion and Resistance to Direct-Acting Antiviral (DAA) Agents

Cell-to-cell infection persists even in the presence of anti-HCV monoclonal antibodies or immunoglobulins, suggesting that this infection route is an effective strategy for the virus to escape host humoral immune responses. A study using chimeric viruses demonstrated that seven major HCV genotypes can efficiently utilize this immune escape strategy [129]. This finding demonstrated that cell-to-cell spread plays a key role in viral persistence, by allowing HCV to evade neutralizing antibodies. Furthermore, studies that investigated the phenotype of DAA-resistant HCV strains using the most updated model systems showed that cell-to-cell transmission is the main route of virus spread for DAA-resistant HCV. Thus, cell-to-cell spread of DAA-resistant HCV can result in viral persistence, thereby hampering virus eradication. Conversely, blocking cell-to-cell transmission was shown to be highly effective in inhibiting dissemination of resistant viruses in a cell culture model [130], leading to rapid virus elimination. Thus, these findings demonstrate a central role for cell-to-cell spread in the dissemination and maintenance of drug-resistant variants. They also suggest that blocking cell-to-cell spread of the virus may prevent the emergence of drug resistance during persistent virus infection. 

## 5. Conclusions and Future Perspectives

In recent years, extensive studies have been performed to unravel the molecular mechanisms of HCV entry—however, these studies were not sufficient to provide a complete understanding of virus entry mechanisms [131,132], nor are they sufficient to address the cure for HCV. Because additional HCV entry factors are being identified, it is essential to describe the complete and detailed process of HCV entry and construct a systematic entry network. This review described each step of HCV entry into hepatocytes in detail, from particle delivery to hepatocytes, the capture of virus particles upon encountering the first host factor, CD81 binding, complex internalization, and lastly, the fusion and release of viral genome (Figure 1). Additionally, a systematic regulatory network for HCV entry is presented in this review, in which EGFR, a previously-identified HCV entry factor, along with TGFβ-R, are recognized as the upstream central regulators. As illustrated in Figure 2, EGFR and TGFβ-R regulate entry events in an orderly and continuous manner, enabling HCV to enter hepatocytes from the extracellular space and successfully release its RNA genome. This review also described HCV’s ability to hijack several host regulatory pathways to subvert host cells, while downregulating host inflammation and immune responses, to achieve effective persistent infection. It also discussed the critical role of cell-to-cell spread in chronic HCV infection and virus resistance to DAA. Further, this review summarized the role of SR-BI, CLDN1, as well as human CD81 and OCLN as the minimal set of HCV entry receptors. 

Based on the current state of knowledge of HCV entry-related factors and regulation, future studies on HCV entry and therapeutic targets should address the following three aspects. (1) c-Cbl may selectively ubiquitinate one of HCV co-receptors in the early step of virus internalization. Thus, end-labeling of specific receptors that activate the subsequent entry processes can be performed to precisely identify the effectors based on the protein mass. In the case that the disruption or inhibition of such ubiquitination abrogates HCV infection, the factor may be a potential therapeutic target for HCV therapeutics. (2) The NF-κB and SMAD6/7 pathways are associated with inflammatory and immune responses, and inhibitory feedback regulations exist for both pathways. These pathways may not appear to benefit the initial HCV entry; however, they may play a role in the chronic maintenance of HCV, contributing to the inability of the host to fully clear the virus. Additionally, inhibition of these two critical pathways may be a strategy to achieve a cure for HCV. (3) Cell-to-cell spread is harmful for the host—this infection route enables the virus to escape host humoral immune responses, leading to chronic infection. Thus, blocking cell-to-cell spread may also serve as a therapeutic strategy for HCV.
